# Leading with humility: unpacking employee career adaptability

**DOI:** 10.3389/fpsyg.2026.1839157

**Published:** 2026-05-22

**Authors:** Michelle Murray, Ekin K. Pellegrini

**Affiliations:** University of Missouri-St Louis Ed G Smith College of Business, St. Louis, MO, United States

**Keywords:** career adaptability, humble leadership, LMX, mentoring, talent management

## Abstract

**Introduction:**

Humble leadership and internal talent management are both significant areas of interest in management research. Yet, little is known about their combined association with employee career adaptability, a key resource in an increasingly competitive human capital landscape. Using social exchange theory, we study the mediating roles of leader-member exchange (LMX) and mentoring in the relationship between leader-expressed humility and employee career adaptability, and the moderating impact of internal talent mobility programs (ITM).

**Methods:**

Data were collected from 178 employees across organizations in the U.S.

**Results:**

Results showed that leader humility was positively related to LMX and mentoring, and mentoring was positively associated with employee career adaptability. Our analysis revealed a marginal moderating impact of ITM, suggesting that a comprehensive ITM platform may amplify the positive association between mentoring and employee career adaptability.

**Discussion:**

Theoretically, we advance the humble leadership literature by positioning supervisory mentoring as the mechanism translating leader-expressed humility into career adaptability. Practically, results highlight the necessity for organizations to elevate talent management and internal mobility as strategic priorities to build resilient talent pipelines. Ultimately, this research underscores that employee adaptability is not solely an individual capability, but is relationally and contextually shaped within the organizational environment.

## Introduction

*Humility is the foundation of all virtues*. — Saint Augustine (c. 410/1990), (n.d.).

With growing media awareness on corporate misconduct, scholarly interest in leadership virtues such as humility has increased within the last decade (Ou et al., 2018; Owens et al., 2019). Previous research has shown leader humility may positively influence information sharing, shared leadership, follower moral behaviors, organizational citizenship behaviors, employee performance, team psychological safety, team creativity, and team performance (Chen et al., 2021; Chiu et al., 2016; Cho et al., 2020; Edmondson, 2018; Hu et al., 2018; Oc et al., 2015; Owens and Hekman, 2015; Owens et al., 2019; Rego et al., 2020). Ou et al. (2024) found humble CEOs may reduce corporate social irresponsibility occurrences. Further, humble leaders may positively impact organizational culture, team humility, and employee voice (Chandler et al., 2023; Chintakananda et al., 2024; Peng et al., 2020; Rego et al., 2017; Zou and Chen, 2022; Chaudhary, et al., 2023).

As research on humble leadership continues to flourish, there are numerous calls for research. Kelemen et al. (2023) suggest research on the impact of mentors in developing humble leaders. Peng et al. (2020) suggest when leaders model humble behavior, there may be a trickle-down effect to lower-level leaders. Qin et al. (2019) called for research on leader humility and subordinate outcomes by studying the role of leader-member exchange (LMX). Further, previous research suggests mentoring and LMX are distinct yet complementary constructs and often act together to enhance employee outcomes (Scandura and Schriesheim, 1994). Based on previous calls for research, this study extends research on leader humility by studying its relationship with LMX, mentoring, and employee career adaptability to inform business organizations as they continuously upskill their workforce to navigate complex and dynamic business environments.

In addition, organizations are increasingly adopting internal talent mobility strategies, such as Mastercard’s *Unlocked* which refers to unlocking the potential of employees within an internal talent marketplace (Bersin and Enderes, 2022). This study examines the moderating impact of an effective internal talent mobility platform on advancing employee career adaptability in an increasingly competitive human capital landscape.

Using social exchange theory as our theoretical foundation, we study humble leaders through their social interactions, such as LMX and mentoring relationships (Lehmann et al., 2023). Qin et al. (2019) suggest leader humility may be positively associated with LMX when employees view that the leader is not acting in a self-serving manner. Humble leaders may act as role models and mentor employees (Lemoine et al., 2019; Rego et al., 2017). However, despite the growing research on humble leadership, research on humble leadership and mentoring has been scant (Chan et al., 2024; Kelemen et al., 2023).

**Figure 1:**
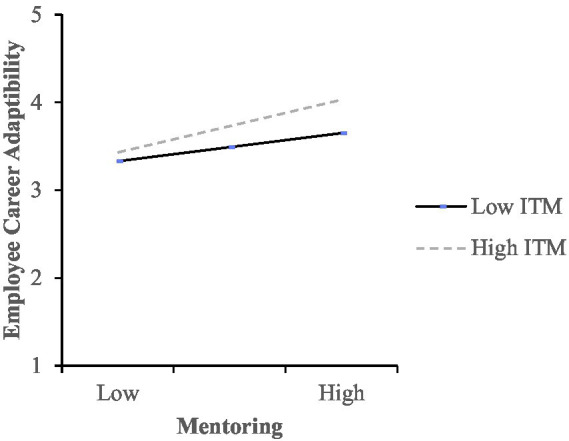
Research model.

This study makes three meaningful contributions to research on humble leadership and career adaptability. First, we examine the relationship between leader humility, LMX and mentoring from the employee’s perspective. Second, to our knowledge, this is the first study to examine the relationship between mentoring and career adaptability. Third, we examine the moderating impact of internal talent mobility practice on the relationship between mentoring and career adaptability for a more comprehensive understanding of the talent management dynamics that impact career adaptability (see Figure 1). Despite the rise of internal talent mobility practices, academic research has lagged behind and this is the first study to empirically examine the return on investment of these platforms on enhancing supervisory mentoring outcomes.

### Humble leadership

Humility has been studied both as a behavior and an innate trait with both intrapersonal and interpersonal dimensions. Owens et al. (2013) defined expressed humility as the ability to view oneself accurately, appreciation of others’ strengths and contributions, and teachability which represents the interpersonal aspect that arises in social contexts (Chandler et al., 2023; Owens and Hekman, 2012; Chandler et al., 2023). Oc et al. (2015) identified nine aspects of humble leadership: possessing an accurate self-view, recognizing follower strengths and accomplishments, modeling teachability and receptiveness to feedback, leading by example, exhibiting modesty, collaborating for the collective good, demonstrating empathy and being approachable, displaying mutual respect and fairness, and acting as a mentor and coach. Ali et al. (2021) also defined humble leadership as appreciating the qualities of others and being open to criticism and new ideas. On the other hand, Ou et al. (2014) referred to humility as a stable interpersonal trait and defined humility through a transcendent self-view as the cognitive core and low self-focus and self-transcendence pursuit as the motivational drive. Additionally, a study by Petrenko et al. (2019) defined humility as a stable personality trait and found that analyst expectations for earnings per share may be lower for organizations with humble CEOs. Leader humility also has been described as a bottom-up leadership style that includes listening, observing others, and hands-on learning (Zhou and Wu., 2018).

In this study, we refer to humble leaders as individuals who possess an accurate self-view, respect all team members, seek suggestions, accept feedback, admit mistakes and lack of knowledge, and want to learn (Ali et al., 2020; Argandona, 2015; Fei and Wu, 2018; Kelemen et al., 2023; Ou et al., 2018). Gaining a deeper understanding of leader humility and its nomological network is informative not only for leaders and employees, but also for business organizations and industries competing in an increasingly dynamic global environment (Cuenca et al., 2022; Nielsen and Marrone, 2018; Lehmann et al., 2025).

Humble leadership may seem similar to related leadership constructs, such as authentic leadership, servant leadership, and ethical leadership; however, each is unique and distict from one another (Lemoine et al., 2019; Maldonado et al., 2022). Authentic leaders can develop followers into leaders through role modeling, however, authentic leaders are centered on self, their values, and beliefs and convey to employees what is required for individual, team, and organizational performance while humble leaders seek feedback and contributions of others (Avolio and Gardner, 2005). Although there is overlap as both authentic leadership and humble leadership focus on self-awareness and modeling behavior to followers, authenticity does not imply humility (Kelemen et al., 2023). Since authentic leadership is about being true to oneself, one may view themselves as superior to others whereas humble leaders seek to accurately assess and recognize both their capabilities and limitations (Kelemen et al., 2023).

Humble leadership and servant leadership are both moral-oriented leadership constucts, however whereas servant leadership includes stewardship and models serving others, humble leadership models the process of becoming and teachability (Ali et al., 2020; Eva et al., 2019; Luo et al., 2022; Owens and Hekman, 2012). Humble leadership involves self-awareness and recognizes one’s strengths and weaknesses while servant leadership does not include self-awareness as part of its definition (Kelemen et al., 2023).

Ethical leadership is focused on compliance with normative standards whereas humble leadership is not focused on compliance, integrity or ethical standards (Lemoine et al., 2019; Thiel et al., 2018). Although humble leadership can result in compliance with normative standards, research has shown that humble leadership may also result in unethical behavior (Kelemen et al., 2023; K. B et al., 2021).

### Leader-expressed humility

Owens et al. (2013) refer to expressed humility as a pattern of behaviors in interpersonal interactions that can be observable by others. Expressed humility consists of three interpersonal characteristics: view oneself objectively; appreciate the unique strengths and contributions of others; and teachability which includes receptiveness to new ideas and feedback (Owens et al., 2015; Rego et al., 2020).

Consistent with the diverse definitions of humility adopted in prior research, previous studies have investigated multiple dimensions of leader humility using a range of measurement instruments. For example, Ou et al. (2014) studied CEO humility using both cognitive and motivational components extending the definition of humility to reflect transcendent self-views and pursuits. Oc et al. (2015) studied leader humility in Singapore, and identified nine dimensions of leader humility of which five were unique to Singapore (i.e., leading by example, showing modesty, empathy and approachability, working together for the collective good, mentoring and coaching) while the other four were consistent with the Owens et al. (2013) conceptualization of leader humility. The numerous conceptualizations of leader humility in previous studies reveal similarities in its core definition across studies, which include viewing self accurately, recognizing the contributions of others, and teachability (Kelemen et al., 2023; Owens et al., 2013).

### Leader-member exchange

LMX theory focuses on the unique relationship that develops between the leader and each follower (Graen and Uhl-Bien, 1995). High quality LMX refers to an “in-group” characterized by affect, loyalty, respect, and mutual contributions which are reflective of social exchange relationships, while low LMX refers to an “out-group” indicative of a transactional or economic exchange relationship (Cropanzano et al., 2017; Liden and Maslyn, 1998).

Research has shown that high quality social exchange may reduce workplace conflict and improve knowledge sharing and employee performance (Cropanzano et al., 2002; Mitchell et al., 2012). Previous research has shown that when employees do not attribute leader humility to a self-serving purpose, leader humility may be positively related to LMX quality (Qin et al., 2019). Dasborough and Ashkanasy (2002) suggested that when leader’s behavior is perceived as sincere and not self-serving, LMX may increase. Wang et al. (2018) found that humility may increase leader’s relational energy which may stimulate high-quality relationships with followers. Therefore, we expect that leader-expressed humility will be positively related to LMX.

*Hypothesis 1:* Leader-expressed humility is positively related to LMX.

### Mentoring

Previous research suggests mentoring and LMX frequently operate together to enhance career outcomes (Scandura and Schriesheim, 1994). Mentoring is an interpersonal relationship between the mentor, a more experienced employee, and the mentee providing career support, psychosocial support, and role modeling (Kram, 1983; Scandura and Ragins, 1993). Career support functions promote career advancement and include sponsoring, coaching, providing challenging assignments, exposure and visibility while psychosocial support functions include counseling and friendship. Role modeling refers to the processes where the protégé respects and emulates the mentor (Allen et al., 2017; Kram, 1983).

Organizations create formal mentoring programs as a talent management tool to develop a leadership pipeline, however, formal mentoring between the mentor and mentee tends to be short-term (Inzer and Crawford, 2005; Joo and Cruz, 2023). On the other hand, informal mentoring relationships are voluntary, evolve organically, and tend to grow into longer-term mentoring relationships (Inzer and Crawford, 2005; Ragins and Cotton, 1999). Holt et al. (2016) suggest that when employees are already in formal mentoring relationships, they may be more likely to seek informal mentoring relationships when LMX quality with their mentor is low.

Although previous studies have shown leader humility to be positively related to the development and engagement of employees, research is still scarce on the relationship between leader humility and mentoring (Kelemen et al., 2023; Lehmann et al., 2023). Humble leaders model behavior and send social cues to employees, and this messaging may be relayed through informal mentoring emerging through high-LMX relationships (Lehmann et al., 2023). Research has shown that LMX may focus on providing positional resources for short-term career outcomes, such as good performance ratings whereas mentoring may be more developmental and related to longer-term outcomes, such salary and promotion (Scandura and Schriesheim, 1994). Social exchange theory (Blau, 1964) suggests that social exchange relations, such as LMX, involve unspecified benefits like developmental assignments. Drawing on social exchange theory, we suggest high-quality LMX relationships represent the primary conduit through which social exchange processes unfold, increasing the likelihood that leaders invest in subordinates’ development. Within this social exchange framework, mentoring emerges as a form of reciprocation and resource provision, through which leaders offer career-related guidance, psychosocial support, and developmental opportunities. These mentoring functions, in turn, equip employees with the resources necessary to enhance their career adaptability. Accordingly, LMX and mentoring constitute theoretically distinct yet sequentially ordered mediators that transmit the effects of humble leadership on employee career adaptability. Therefore, we expect quality of LMX to be positively related to mentoring.

*Hypothesis 2:* LMX is positively related to mentoring.

### Employee career adaptability

Career adaptability refers to resources that assist an individual to effectively manage career-related work challenges, transitions, and adapting to a changing work environment (Savickas and Porfeli, 2012). It reflects personal agency and the ability to mindfully devise adaptive behaviors and career strategies (Haenggli and Hirschi, 2023; Hamtiaux et al., 2013; Peng et al., 2021; Savickas and Porfeli, 2012; Spurk et al., 2020). In their meta-analysis, Rudolph et al. (2017) found career adaptability to offer numerous benefits to employees, including job satisfaction, organizational commitment, promotability, income, performance, and reduced stress. Previous research has found employee career adaptability to be a critical resource that enable organizations to quickly and effectively refine processes, respond to events timely, engage the workforce, and reduce turnover (Chughtai et al., 2023; Ployhart and Bliese, 2006).

Career adaptability is a multimensional construct and involves concern, control, curiosity, confidence, and cooperation (Savickas, 2013; Prasad et al., 2021). *Concern* refers to efforts to prepare for career experiences, such as planning to achieve goals. *Control* refers to individuals taking responsibility for shaping their career, such as taking responsibility for actions. *Curiosity* is the ability to envision various roles, such as exploring career possibilities. *Confidence* refers to feeling capable of tackling career challenges, such as overcoming obstacles, and *cooperation* refers to the ability to work well with others, such as getting along with team members (Nye et al., 2018).

Organizations are increasingly impacted by internal and external factors that affect financial performance, such as technological advancements, political and economic factors, and employee career adaptability is crucial for organizations to quickly and effectively tackle emerging challenges and capitalize on emergent opportunities (Haibo et al., 2017; Neves and van Dam, 2024; Tariq et al., 2011). In this context, mentoring may be a tool that encourages receptiveness to learning, and guides employees in their careers (Chughtai et al., 2023). As the workplace continues to evolve, it is important to have a comprehensive understanding of how employee career adaptability can be cultivated. The positive effects of leader humility on key employee outcomes have been well documented, however less attention has been given to the theoretical mechanisms that explain how and when leader expressed humility impacts desired employee outcomes, such as career adaptability. Extending prior research and based on SET, we expect that mentoring is positively related to employee career adaptability.

*Hypothesis 3:* Mentoring is positively related to employee career adaptability.

Integrating social exchange theory, we propose a sequential mediation mechanism linking humble leadership to employee career adaptability through LMX and mentoring. Humble leadership fosters high-quality LMX relationships, which in turn, create a relational foundation to engage in mentoring behaviors as a form of social exchange. Mentoring then serves as the proximal mechanism through which employees receive developmental resources that enhance their career adaptability. Therefore, we expect LMX and mentoring to jointly operate as sequential mediators in this relationship.

*Hypothesis 4:* LMX and mentoring sequentially mediate the relationship between humble leadership and employee career adaptability.

### Internal talent mobility

Research suggests numerous positive outcomes, such as employee engagement and organizational commitment, when employees perceive their organization is supportive of their career growth and invest in talent development (Jiang et al., 2023; Rigolizzo, 2019; Zacher, 2015). Recently, there is an emerging organizational practice of talent cultivation from within the organization that is referred to as the internal talent marketplace that support employee learning, career growth, and talent mobility within the organization (Cowgill et al., 2023; Williamson and Carroll, 2023). The internal talent marketplace promotes employee internal mobility which provides organizations with enhanced performance, value creation, and innovation (Bibi, 2024).

The organizational internal talent marketplace is a digital platform designed to strategically align employees with relevant trainings, projects, job assignments, and job openings, and it not only allows organizations to recognize employees’ potential but may also lower costs associated with external recruitment, shorten the hiring process, and reduce employee turnover (Bersin and Enderes, 2022; Bibi, 2024; Cowgill et al., 2023). Companies such as Mastercard, Google, Booz Allen, Department of Defense, and Teach for America have successfully implemented internal talent marketplaces with employees reporting feeling more empowered and enhanced connections across the organization which in turn improve employee retention (Williamson and Carroll, 2023). Mastercard refers to their internal marketplace program as *Unlocked*, meaning the practice was implemented to help unlock the potential of employees.

Internal talent mobility (ITM) platforms represent a rapidly emerging organizational practice in contemporary digital talent management systems. As organizations increasingly adopt platform-based approaches to internal mobility and skill development, it becomes theoretically and practically essential to examine their implications for established developmental mechanisms such as mentoring. We propose that the effectiveness of mentoring in fostering career adaptability is contingent upon the organizational context in which developmental exchanges are embedded. Career construction theory emphasizes that career adaptability is developed not only through access to such resources, such as mentoring, but as critically, through their active enactment in relevant career experiences. In this regard, ITM serves as a critical structural enabler that facilitates the translation of mentoring-derived resources into adaptive career outcomes by offering transparent access to project assignments and internal mobility opportunities for employees to apply and refine the insights and skills gained through mentoring.

Given that the mentoring-career adaptability relationship remains relatively untested, explaining any significant relationships is critical. In addition, studying the moderating impact of ITM enables scholarly research to remain aligned with evolving organizational practices and to provide timely insights that can inform both theory and practice. A comprehensive ITM platform may capture the extent to which the organizational environment enables the conversion of mentoring into career adaptability, thereby strengthening this relationship when developmental opportunities are more visible, accessible, and actionable. Therefore, we expect that the positive relationship between mentoring and employee career adaptability will be augmented in organizations high (vs. low) levels of comprehensive internal talent mobility programs.

*Hypothesis 5:* The positive relationship between mentoring and career adaptability is stronger in organizations with a more comprehensive internal talent mobility platform, and weaker when such a platform is less developed.

## Methods

### Procedures and participants

Participants completed an online survey through Prolific that included screening questions to verify that they met the inclusion criteria. To meet the study inclusion criteria, participants were at least 25 years old, employed full-time, reside and work in the U.S., have at least 5 years of full-time work experience providing participants with a sufficient opportunity to have developed a mentoring relationship in a full-time work environment, and reporting to their direct supervisor for at least one year. Participants were asked to evaluate their relationship with their direct supervisor, and the minimum one-year requirement provides employees with an adequate timeframe to be able to meaningfully evaluate their relationship with their supervisor. All participants were employed at companies with at least 500 employees so that the companies may be more likely to have implemented an internal talent mobility marketplace.

A total of 198 participants both met the inclusion criteria and completed the survey through Prolific. To ensure that participants allocated sufficient time to thoughtfully respond to survey questions, Z-scores were calculated for total survey completion time. The distribution of response times was right-tailed which indicated some respondents took an unusually long time to complete the survey. The 198 survey participants had a mean completion time of 11.11 min and Z-scores ranged from −0.91 to 5.05. Twenty participants took 21.87 to 57.62 min to complete the survey. Survey completion times which exceeded ±3 Z-score deviations from the mean were identified as outliers and removed. The mean and Z-scores were recalculated, and additional responses with completion times exceeding ±3 Z-score deviations from the recalculated mean were removed to ensure participants were focused during survey completion. After this screening process, the final sample included 178 participants. The mean completion time was 8.49 min (SD = 3.93).

Multivariate outlier assessment was conducted using Mahalanobis distance (Tabachnick and Fidell, 2019) with the full set of study variables. Mahalanobis distance values were compared against the chi-square distribution with degrees of freedom equal to the number of variables at the *p* < 0.001 threshold (*χ*^2^(df) = 114.835). Five cases exceeded the critical value of 114.835. Following Kline (2016), these five cases were evaluated for statistical extremity and practical influence on the model. The identified cases did not show undue influence on the overall model fit, and they were retained in the final analyses. Mahalanobis *D*^2^ histograms were also evaluated. The distribution of *D*^2^ scores appeared normal with *N* = 178, with most cases clustering below the critical threshold and the decision was made to retain these five cases since they may represent valid observations within the population rather than anomalies.

The age ranges with the highest representation were 31 to 40 (44.4%), and 41 to 50 (22.5%). The sample included 105 women (59%) and the majority of the sample (76%) reported holding a bachelor degree or higher. Regarding full-time work experience, 31.5% have worked for 10 to 19 years, 28.1% for 5 to 9 years, 24.2% for 20 to 29 years, and 16.3% for over 30 years. Respondents reported their ethnicity as White (65.7%), Black or African American (18.0%), Hispanic or Latino (9.0%), and Asian (7.3%). Participants were based in the U.S. and identified their region as South (35.4%), Midwest (22.5%), West (21.9%), and Northeast (20.2%).

### Measures

*Leader-expressed humility* was measured using the 11-item scale developed by Owens et al. (2015). Sample items include “My direct supervisor admits it when they don’t know how to do something” and “My direct supervisor takes notice of others’ strengths.” Items were rated on a 5-point scale ranging from 1 (Strongly Disagree) to 5 (Strongly Agree). The internal consistency of scale scores was 0.95. The overall mean was 3.84 (SD = 0.785), mode was 4.00, and the range of scores was 1 to 5. Skewness was −1.12 and kurtosis was 1.39, both within acceptable ranges for normality (Kline, 2016). Shapiro–Wilk test indicated a normal distribution (*W* = 0.917, *p* < 0.001). A confirmatory factor analysis (CFA) was conducted to assess unidimensionality of the scale scores. All items in the loaded significantly on the latent factor, with standardized loadings ranging from 0.50 to 0.85. The Average Variance Extracted (AVE) was 0.72, exceeding the recommended threshold of 0.50 (Fornell and Larcker, 1981). Composite Reliability (CR) was 0.97, supporting construct validity.

*Leader-member exchange* (LMX) was assessed using the 12-item multidimensional LMX scale (LMX-MDM) developed by Liden and Maslyn (1998). This scale measures four LMX dimensions: affect, loyalty, contribution, and professional respect. Sample items include “I like my supervisor very much as a person.” and “I admire my supervisor’s professional skills.” Responses were on a 5-point scale and the internal consistency of scale scores was 0.94. The overall mean was 3.74 (*SD* = 0.787), the mode was 3.33, and the range of scores was 1.17 to 5. Skewness was −0.757 and kurtosis was 0.483, both within acceptable ranges for normality. Shapiro–Wilk test supported normality (*W* = 0.956, *p* < 0.001). A CFA was conducted to assess the factorial structure of the LMX-MDM scale scores. All items loaded significantly on their respective dimensions with standardized loadings ranging from 0.28 to 0.83; the low loading of 0.28 was for item 7. With item 7 included, the AVE was 0.58 and the CR was 0.95, supporting the construct’s convergent validity and excluding the low loading item 7, the AVE would have improved to 0.62 and CR to 0.96, slightly improving model fit.

*Mentoring* was assessed using the 9-item Mentoring Functions Questionnaire (MFQ-9) developed by Castro and Scandura (2004). Sample items include “My direct supervisor takes a personal interest in my career” and “My direct supervisor helps me coordinate professional goals.” Responses were on a 5-point scale and the internal consistency of scale scores was 0.95. The overall scale score mean was 3.25 (*SD* = 0.977), the mode was 3.44, and the range of scores was 1 to 5. Skewness was −0.47 and kurtosis was −0.67, both within acceptable ranges for normality assumptions (Kline, 2016). Shapiro–Wilk test supported normality (*W* = 0.95, *p* < 0.001). A CFA was conducted to assess the factorial structure of the MFQ-9 scale scores. All items loaded significantly on their respective dimensions with standardized loadings ranging from 0.70 to 0.88. The AVE was 0.64, and CR was 0.95, both exceeding established thresholds, supporting convergent validity.

*Career adaptability* was assessed using the 30-item scale developed by Nye et al. (2018) which includes five subscales: Control, Curiosity, Concern, Confidence, and Cooperation. Sample items include “Making decisions by myself” and “Learning new skills.” Respondents rated how strongly they had developed the abilities on a 5-point scale ranging from 1 (Not Strong) to 5 (Strongest). The overall internal consistency of scale scores was 0.93. Subscale means ranged from 3.51 (Concern, *SD* = 0.62) to 3.83 (Confidence, *SD* = 0.90). The scale demonstrated very good internal consistency, with a Cronbach’s alpha of 0.93. CR was 0.93, which exceeded the threshold. The overall scale mean was 3.63 (*SD* = 0.572), the mode was 3.77, and the range of scores was 1 to 5. Skewness was −0.18 and kurtosis was 0.28, both within acceptable ranges for normality. Shapiro–Wilk test supported normality (*W* = 0.991, *p* = 0.348).

A CFA with all items was conducted and all items loaded on their respective factors, with standardized loadings ranging from 0.23 to 0.85. The AVE and CR for each subscale were examined and all AVE values exceeded the recommended threshold of 0.50, all CR values were above 0.70, suggesting the scale’s convergent validity. For the overall scale, the exclusion of a few items would slightly improve reliability indices, however the minimal improvement in fit led to the decision not to remove any items for construct validity.

*Internal talent marketplace* was measured using a 10-item scale developed for this study. Participants were provided with the statement “An internal talent marketplace is a company-run internal platform where employees create an online profile with information on their background, skills, and interests” and were asked to rate their responses on a 5-point scale reflecting on their current organization. Sample items include “My organization’s internal talent marketplace provides access to short-term projects”, “My organization’s internal talent marketplace offers personalized learning recommendations designed to match career development aspirations”, and “I find my company’s internal talent marketplace to be helpful for my career.” An exploratory factor analysis (EFA) using principal axis factoring with oblique rotation supported a unidimensional structure, with all items loading above 0.79. Cronbach’s alpha was 0.97, indicating excellent internal consistency. The scale mean was 2.80 (*SD* = 1.18), the mode was 1.00, and the range of scores was 1 to 5. Skewness was −0.18 and kurtosis was −1.2, both within acceptable ranges for normality assumptions. Shapiro–Wilk test indicated a normal distribution (*W* = 0.929, *p* = <0.001).

*Self-serving leadership* was used as a control variable as it may create an environment with lower psychological safety and less knowledge sharing (Peng et al., 2018). Self-serving leadership was assessed using the 4-item scale by Camps et al. (2012). The scale assesses employees’ perceptions of their leader engaging in behaviors that prioritize personal interests. Responses were on a 5-point scale and a sample item includes “My supervisor would forge a document when this could improve his/her position.” Cronbach’s alpha was 0.88. Item-deletion analysis revealed that all items contributed meaningfully to internal consistency. CFA results supported the unidimensionality of the scale, with all items loading significantly on the latent factor. Standardized loadings ranged from 0.68 to 0.93. The AVE was 0.66, and CR was 0.88, supporting convergent validity. The overall scale mean was 1.88 (*SD* = 0.902), and the mode was 1.00. The range of scores was 1.00 to 5.00. Skewness was 1.16 and kurtosis was 0.93, indicating a normal distribution. Shapiro–Wilk test indicated a normal distribution (*W* = 0.862, *p* < 0.001).

To ensure that Common Method Variance (CMV) did not unduly influence our results, we proactively employed a multi-faceted approach consisting of a marker variable technique and Harman’s Single-Factor test. To rigorously test for method effects, we embedded two marker variables into our survey design that are theoretically unrelated to our primary study constructs.

The *Attitude Toward the Color Blue* scale by Miller et al. (2024) was used as a marker variable. This 4-item scale is theoretically unrelated to the study constructs and was included to provide an empirical test for CMV using the marker variable technique (Podsakoff et al., 2003). Responses were on a 5-point scale ranging from 1 (Strongly Disagree) to 5 (Strongly Agree). A sample item is “Blue is a nice color.” As expected, the zero-order correlations between this marker variable and the study variables were statistically nonsignificant. Further, following the marker variable adjustment approach, partial correlations were calculated controlling for the marker variable, with no substantive change in the pattern, significance, or strength of the hypothesized relationships.

*Daily Mood* was assessed as an additional CMV marker, using the 6-item scale by Kouros and El-Sheikh (2015). This scale evaluates affective states using bipolar semantic differential items anchored from 1 to 5 (e.g., calm [5] vs. jittery [1]), and it is theoretically unrelated to the study constructs. Participants were asked to select the response that best represented their typical emotional state during the day. Similarly, the zero-order correlations between the Daily Mood marker variable and the primary study variables were all statistically nonsignificant. Partial correlations were calculated controlling for daily mood, with no substantive change in the pattern, significance, or strength of the primary relationships suggesting that respondents’ general affect or disposition did not systematically inflate our construct relationships.

In addition to the marker variable approach, Harman’s single-factor test was conducted using an EFA on all study variables, including the two marker variables. The first (largest) factor accounted for 28.6% of the total variance, well below the 50% threshold used as an indicator of CMV concerns (Podsakoff et al., 2003). Based on the robust evidence from both the partial correlation procedures and the EFA, we are confident that common method bias was unlikely to pose a threat to the validity of the study’s results.

## Results

Descriptive statistics, reliabilities, and correlations are presented in Table 1. Consistent with expectations, leader-expressed humility, mentoring, and LMX were significantly and positively correlated with career adaptability. Self-serving leadership showed significant negative associations with all study variables. We calculated the Variance Inflation Factor (VIF) and tolerance values for each variable. VIF values ranged from 1.06 to 1.79. All tolerance values exceeded 0.56. According Hair et al. (2020), VIF values below 5.0 and tolerance values above 0.20 indicate that multicollinearity is not a concern and that our model estimates are stable.

**Table 1:** Means, standard deviations, and intercorrelations among variables (*N* = 178).

Variable	M	SD	1	2	3	4	5	6
1. Leader-expressed humility	3.84	0.79	0.95					
2. Leader-member exchange (LMX)	3.74	0.79	0.83**	0.94				
3. Mentoring	3.25	0.98	0.77**	0.87**	0.95			
4. Career adaptability	3.63	0.57	0.28**	0.42**	0.42**	0.93		
5. Internal talent marketplace (ITM)	2.80	1.18	0.15**	0.19**	0.33**	0.36**	0.97	
6. Self-serving leadership	1.88	0.90	−0.64**	−0.65**	−0.56**	−0.16**	0.07	0.88

**p < 0.01; Cronbach’s alpha coefficients are presented on the diagonal.

Hypothesis 1 proposed a positive relationship between leader-expressed humility and LMX. We tested these effects with leader-expressed humility as the independent variable, and self-serving leadership, age, work experience, and gender entered as control variables, with LMX as the dependent variable. The model was statistically significant [*F*(5, 172) = 89.40, *p* < 0.001]. *R*^2^ was 0.72 and the effect size was 2.60, which represents a large effect (Cohen, 1988). To assess the relative importance of each predictor, a relative weight analysis (RWA) was conducted, which decomposes the model’s *R*^2^ into weights that reflect the proportion of variance uniquely attributable to each predictor, accounting for both its direct effect and its shared variance with other predictors (Tonidandel and LeBreton, 2011). Leader-expressed humility accounted for the largest proportion of explained variance, contributing 68.0% of the total *R*^2^. This provides additional evidence of its association beyond statistical significance alone. Although leader-expressed humility was the most significant predictor of LMX, as expected, self-serving leadership also had a significant negative effect suggesting that self-serving leadership may negatively relate to LMX. Results provide support for Hypothesis 1, suggesting that employees who perceive their supervisors as exhibiting humility may be more likely to experience a higher quality LMX with their supervisor.

Hypothesis 2 suggested a positive relationship between LMX and supervisory mentoring. We tested these effects with LMX as the independent variable, and self-serving leadership, age, work experience, and gender entered as control variables, with mentoring as the dependent variable. The overall model was statistically significant [*F*(5, 172) = 112.45, *p* < 0.001]. *R*^2^ was 0.77 and the effect size was 3.27 which represents a large effect (Cohen, 1988). LMX emerged as a significant predictor of supervisory mentoring, accounting for the largest proportion of explained variance, contributing 76.7% of the total *R*^2^. Results provide support for Hypothesis 2 and suggest employees who experience high quality LMX with their supervisor may also be more likely to perceive higher levels of mentoring from their supervisor.

Hypothesis 3 proposed a positive relationship between mentoring and employee career adaptability. We tested these effects with mentoring as the independent variable, and self-serving leadership, age, work experience, and gender entered as control variables, with career adaptability as the dependent variable. The overall model was statistically significant [*F*(5, 172) = 8.27, *p* < 0.001]. *R*^2^ was 0.19 and the effect size was 0.24. Mentoring accounted for 87.2% of the total *R*^2^, providing additional evidence of its substantive link beyond statistical significance alone. Results provide support for Hypothesis 3 and suggest employees who receive mentoring from their direct supervisor may be more likely to experience career adaptability.

Hypothesis 4 was tested using Structural Equation Modeling (SEM) analysis in AMOS. The model showed an acceptable fit to the data (*χ*^2^(1804) = 3015.45, *p* < 0.001; *χ*^2^/df = 1.672; RMSEA = 0.06, CFI = 0.86). All structural paths were positive and statistically significant (*p* < 0.001). The results suggest when LMX and mentoring are included in the model, the direct effect of humble leadership on employee career adaptability becomes non-significant, indicating full serial mediation through LMX and mentoring. Further, using a bootstrapping procedure, we found that the serial indirect effect of humble leadership on employee career adaptability through LMX and mentoring was statistically significant. Leader humility had a significant indirect effect on mentoring through LMX (*β* = 0.90, 95% BC CI [0.806, 0.997], *p* < 0.001), indicating that leader humility is linked to mentoring via LMX. Further, leader humility had a significant indirect effect on employee career adaptability through the sequential mediation of LMX and mentoring (β = 0.21, 95% BC CI [0.119, 0.391], *p* < 0.001), supporting full mediation. Results support Hypothesis 4 and suggest that leader humility may enhance employee career adaptability through the mediated pathway of LMX and mentoring.

**Figure 2:**
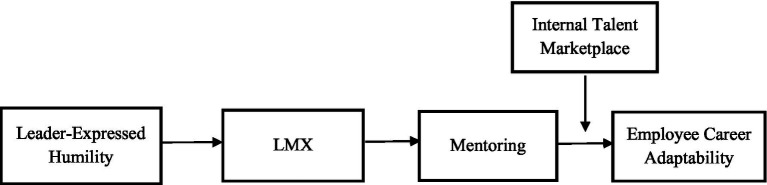
Changes in career adaptability as a function of mentoring and ITM.

Hypothesis 5 suggested the positive link of mentoring on career adaptability would be stronger in organizations with a comprehensive internal talent mobility platform. The overall model was statistically significant [*F*(7,170) = 8.37, *p* < 0.001], with an *R*^2^ of 0.26. The interaction between mentoring and internal talent mobility was significant (*b* = 0.06, *p* = 0.08). Results indicate that an internal talent mobility platform may enhance the positive effect of mentoring on employee career adaptability suggesting that an internal talent mobility practice may amplify the positive impact of mentoring direct supervisors provide (see Table 2). To interpret the interaction, a simple slopes analysis was conducted (see Figure 2). At low levels of internal talent mobility (−1 SD), mentoring was positively associated with employee career adaptability (*b* = 0.16, *p* < 0.001). At mean levels of internal talent mobility, the association was stronger and positive (*b* = 0.24, *p* < 0.001). At high levels of internal talent mobility (+1 SD), the positive association was even stronger (*b* = 0.31, *p* < 0.001). Results suggest a significant marginal moderating impact of internal talent mobility; as employee perceptions of an internal talent mobility platform increases, the positive association of mentoring and employee career adaptability becomes significantly stronger. We interpret this finding as a marginal but theoretically notable and practically relevant trend based on Olsson-Collentine et al. (2019) finding that referring to *p*-values between 0.05 and 0.10 as marginally significant is a deeply entrenched and accepted convention across top-tier social science journals. Pritschet et al. (2016) also defend the utility of reporting marginal effects, noting that adherence to a strict 0.05 cutoff may ignore evidence and cause researchers to discard theoretically valuable trends. Our data suggest when high levels of supervisory mentoring is combined with an organizationally managed comprehensive internal talent mobility platform, the combined impact may be associated with greater employee career adaptability. When employee perceptions of an internal talent mobility platform is low, the relationship between mentoring and career adaptability weakens.

**Table 2:** Results testing the main effects and moderation effects.

Variables	Employee career adaptability			
	*b*	*SE*	*t*	*p*
Mentoring	0.24	0.05	4.38	<0.001
ITM	0.10	0.04	2.70	<0.01
Mentoring × ITM	0.06	0.04	1.76	<0.08
*R* ^2^		0.26		
adj. *R*^2^		0.23		

### Supplementary analyses

In this study, LMX was studied as an overall construct. LMX theoretically represents four subdimensions including affect, loyalty, contribution, and professional respect. We conducted linear regression analyses to probe the relative effects of LMX dimensions on leader-expressed humility. Significant differences were observed across all four dimensions (all *p* < 0.001). Patterns of means showed that responses to leader humility have the strongest influence on LMX-loyalty (*R*^2^ *=* 0.60) followed by LMX-affect (*R*^2^ = 0.67). Relative weight analyses suggested leader-expressed humility explained 70.3% of the variance in LMX-affect and 71.3% of the variance in LMX-loyalty.

Perhaps leader humility is most strongly related to LMX-loyalty because when leaders admit mistakes, ask for input, share credit, and acknowledge others’ strengths, followers feel respected and valued, and strengthen loyalty to the leader who provides such experience. With humble leaders, followers may develop loyalty not out of obligation, but because they want to follow a leader who treats them well, listens, and leads with integrity. Similarly, LMX-affect may increase with humble leadership because humble leaders may come across as genuine rather than distant authority figures, and followers may feel greater affect towards leaders who are approachable and authentic. Further, humble leaders do not position themselves “above” others; they invite collaboration and listen. This lower social distance may increase warmth, which is a primary driver of interpersonal liking.

The differential relationship between humble leadership on LMX dimensions was not part of this study’s research questions. However, our data reveal interesting findings and results may provide a groundwork for future research in further examining the positive impact of leader humility in cultivating high-quality LMX relationships.

## Discussion

We developed and tested a theoretical model that specifies how and when leader-expressed humility is associated with employee career adaptability. The study focused on the employee’s perception of their direct supervisor, specifically examining how perceived leader humility relates to employee career adaptability through LMX and mentoring. The study further examined the moderating impact of a novel variable (i.e., ITM), and found a marginal positive impact of an organization’s internal talent mobility program on employee career adaptability, when combined with supervisory mentoring.

The study contributes a novel lens to inform research on employee career adaptability. Business organizations are increasingly adopting internal talent mobility platforms; however, scholarly research examining the return of this investment has lagged behind. Results suggest internal talent mobility systems may amplify the positive association between supervisory mentoring and employee career adaptability suggesting a promising new avenue of research into this emerging new practice for developing internal talent in business organizations.

Further, in this study, the direct effect of LEH on employee career adaptability was not statistically significant (*p* = 0.616) indicating that LEH links to employee carer adaptability primarily through the sequential mediation of LMX and mentoring. These results support full mediation and emphasize the high-ortance of quality interpersonal relationships through LMX and mentoring for the positive associations of leader-expressed humility to emerge.

The interaction between mentoring and internal talent mobility (ITM) is perhaps the most novel finding of the current study, indicating that the positive effect of mentoring on employee career adaptability may be amplified in business organizations that have invested in a comprehensive ITM program. Therefore, ITM may offer a significant competitive advantage as an organizational resource as employees benefit from both developmental relationships at work and formally managed growth opportunities.

Overall, the proposed model accounted for a substantial variance in LMX (69.9%) and mentoring (75.7%), and a meaningful proportion of variance in employee career adaptability (19.5%) which demonstrated the critical roles of LEH, LMX, and mentoring for employee career adaptability. This finding is consistent with the theoretical foundations of social exchange theory.

Employee career adaptability is crucial for organizations to effectuate operational processes in response to changing micro and macro-economic environments and to keep the workforce engaged (Chughtai et al., 2023; Neves and van Dam, 2024; Ployhart and Bliese, 2006). Career construction theory posits that continuous adaptation to the work environment necessitates career adaptability which provides employees a strategic framework for sustaining themselves in an evolving workplace (Ohme and Zacher, 2015; Savickas and Porfeli, 2012). Our study is the first to show that the financial and human capital investment in internal talent mobility programs may reap sizeable benefits, such as employee career adaptability, and also amplify the impact of other developmental practices at work, such as supervisory mentoring.

### Theoretical implications

This study moves beyond viewing humility as a desirable leader trait to theorizing it as a catalyst that activates relational and developmental processes culminating in employee career adaptability. We answer calls to expand the theoretical landscape surrounding leader-expressed humility and employee career adaptability. By anchoring our model in the theoretical foundations of Social Exchange Theory (SET), this research clarifies the relational and psychological mechanisms through which humble leadership translates into employee outcomes. Overall, this study makes four distinct contributions to the literature.

First, we extend the nomological network of leader-expressed humility by establishing its critical association with high-quality LMX. While prior research has established the value of transformational leadership in fostering LMX when perceived as sincere (Dasborough and Ashkanasy, 2002), our findings advance this paradigm by demonstrating that leader humility specifically acts as a powerful catalyst for relational exchange. Theoretically, this suggests that when leaders model vulnerability and teachability, it triggers a reciprocal social exchange process, leading employees to perceive and engage in stronger, higher-quality LMX.

Second, this study bridges the theoretical gap between the humble leadership literature and mentoring research. Although recent scholarship has theorized that leader humility may foster employee development (Kelemen et al., 2023; Lehmann et al., 2023), empirical evidence linking humility-driven LMX to supervisory mentoring has been scarce. Our findings position supervisory mentoring as a direct behavioral manifestation of high-quality LMX. By doing so, we advance SET by demonstrating that the social capital generated by leader humility is explicitly exchanged and deployed through targeted mentoring behaviors.

Third, we integrate SET with Career Construction Theory (Savickas and Porfeli, 2012) by establishing a novel link between supervisory mentoring and employee career adaptability. While previous literature indicates that leadership and career optimism relate to adaptability (Delle and Searle, 2020), and that mentoring assists career trajectories (Chughtai et al., 2023), our study is the first to empirically validate mentoring’s direct link to career adaptability. We contribute to the adaptability literature (Neves and van Dam, 2024) by demonstrating that relational resources (mentoring) may effectively translate into individual resources (adaptability), allowing employees to better navigate complex role transitions.

Finally, we introduce a recently emerging boundary condition to the development of career adaptability by demonstrating the marginal moderating role of internal talent mobility practices. By examining this moderation, we answer calls to more fully define the nomological network of career adaptability. Theoretically, this finding highlights the interaction between micro-level relational dynamics (mentoring) and macro-level organizational contexts (talent mobility). It suggests that the translation of mentoring into career adaptability is significantly amplified when the broader organizational structure actively signals and supports internal mobility, providing a new theoretical pathway for future research on talent pipelines.

### Implications for practice

Leaders who display humility may positively support employee development and thereby building a talent pipeline for their organization. Employees who perceive leadership as humble and who are not self-serving may be more likely to develop high-quality relationships and receive mentoring which may cultivate career adaptability. As organizations reflect on the composition of their leadership teams, it is important to not only examine the financial contributions and impact but also the intangible and long-term impact of the talent pipeline they develop within the organization.

Organizations committed to continuously improving and developing employee talent should allocate resources to support leadership development in relationship building and enhancing employee career development, both of which may facilitate an organization to more competitively adapt to an ever-evolving micro and macro work environment. As organizations evaluate internal policies and procedures, decision makers should consider the support provided to leadership roles in developing the skills to cultivate high-quality dyadic relationships and nurture organic mentoring connections. This study focused on informal mentoring that develops organically in the workplace; however, organizations may also opt to examine the benefits of formal mentoring programs. Although research has shown that formal mentoring programs tend to be short-term, the formation of a mentoring program may be a viable starting point (Allen et al., 2017). Further, Joo and Cruz (2023) suggested that formal mentoring may improve the quality of a mentee’s informal mentoring network. Therefore, based on the current findings, organizations should recognize and leverage the strategic value of mentoring in strengthening their human capital pipeline.

Further, our research showed that the positive effects of mentoring on employee career adaptability may be stronger in organizations with a comprehensive internal talent mobility program which is the first empirical finding that lends support to these emerging HR practices in business corporations. Employee career adaptability facilitates organizations navigating rapidly evolving business landscapes, enabling them to swiftly and effectively refine processes to respond to internal, local, and global fluctuations (Chughtai et al., 2023; Neves and van Dam, 2024; Peng et al., 2021). To effectively respond to these challenges, organizations may consider implementing an internal talent mobility program to support employees and the organization’s future talent pipeline. An internal talent mobility program may provide employees with skill development tools that enhance job performance and engagement while the organization benefits from growth of employee knowledge, skills and connections (Bibi, 2024). An internal talent mobility program matches employees with job openings and projects, benefiting employees by providing access to new opportunities and benefiting organizations by filling existing positions more swiftly and efficiently, shortening the hiring process and reducing costs associated with external recruitment (Bersin and Enderes, 2022; Cowgill et al., 2023). This study shows the benefits that business organizations may receive from implementing a comprehensive internal talent mobility program.

### Limitations and future directions

Notwithstanding its contributions, there are several limitations of this study. First, although we statistically reduced concerns for common method bias, it remains an inherent concern in self-reported data. Further, cross-sectional design limits causal inferences among the study variables. Further, our study participants were based in the U.S. and future studies should study the impact of ITM in cross-cultural research designs, given ITM is increasingly adopted by multinational corporations (MNCs), such as Google and Mastercard (Williamson and Carroll, 2023) and talent mobility across cultural contexts is common in MNCs (Delacruz et al., 2025).

Finally, our study focused on informal mentoring that develops organically in the workplace, and we studied an overall mentoring construct. Future research could study the differential impact of mentoring functions, such as career support, psychosocial support, and role modeling, to deepen our understanding of the relative importance of each in cultivating career adaptability. Additionally, it would be interesting to examine formal mentoring programs and their impact on employee career adaptability. Given that the mentoring-career adaptability relationship remains relatively untested, explaining any significant relationships is critical.

## Conclusion

Employee career adaptability has become a critical driver of business transformation in an increasingly technology-driven and rapidly evolving landscape. Current results suggest that organizations must prioritize strategic internal talent management and proactively develop their talent pipelines to maximize the return on investment in modern organizational initiatives, such as supervisory mentoring and cultivating employee adaptability. As Kakuzo Okakura stated more than a century ago, “The art of life is a constant readjustment to our surroundings”. The art of survival is no different for business organizations. This study underscores that strategically designed human resource practices play a pivotal role in fostering employee career adaptability, thereby enabling organizations to sustain performance amid ongoing environmental and technological disruption for both current and future talent.

## Data Availability

The original contributions presented in the study are included in the article/supplementary material, further inquiries can be directed to the corresponding author.
